# Checklist of British and Irish Hymenoptera - Evanioidea

**DOI:** 10.3897/BDJ.2.e1116

**Published:** 2014-06-17

**Authors:** Gavin R. Broad, Laurence Livermore

**Affiliations:** † The Natural History Museum, London, United Kingdom

## Introduction

Abbreviations, rationale for species inclusion and additional information are given in the introductory article for the series. This article is the first in a series of 13 checklist sections covering the Hymenoptera of the British Isles. For each genus and species the original combination and a list of synonyms is provided under the heading 'nomenclature'.

A corresponding up-to-date checklist for Evanioidea is maintained on the Hymenoptera of the British Isles site.

**Figure 1.**
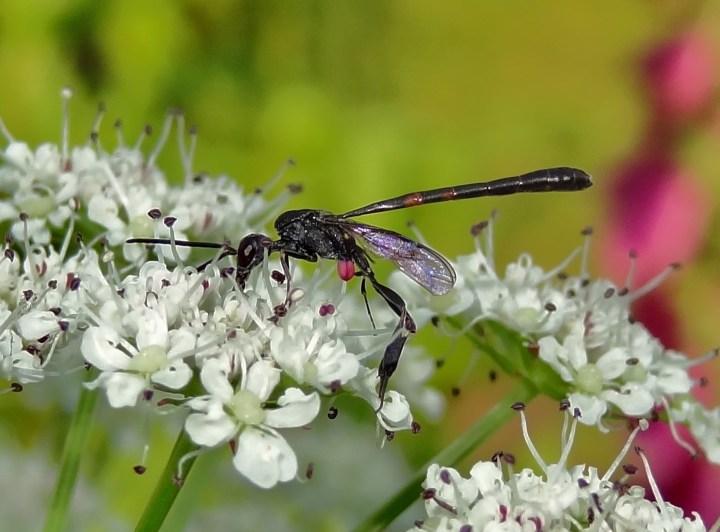
Male *Gasteruption jaculator* (Linnaeus) (Wilmslow, Cheshire) (courtesy of A.M. Broome).

Distribution data for the Evanioidea are taken from Crosskey (1951), with additional references given under notes for each taxon. This is a small superfamily of three extant families, but particularly poorly represented in northern Europe. The Evaniidae (sometimes referred to as 'ensign wasps') are, where known, parasitoids/predators of cockroach oothecae (Crosskey 1951; Brown 1973) and are morphologically very distinct from the remaining families. Whilst rather similar in appearance, the Aulacidae and Gasteruptiidae (Fig. 1) differ markedly in biology, with the single British aulacid, *Aulacus striatus* (Jurine), being a koinobiont endoparasitoid of Xiphydriidae larvae (see E.R. Skinner & W.G. Thompson's 1960 film, 'The Alder Woodwasp and its Insect Enemies', distributed by World Educational Films: http://worldeducationalfilms.com/the-alder-woodwasp-and-its-insect-enemies/) whilst *Gasteruption* species are essentially predators and cleptoparasites of solitary bee larvae (Malyshev 1964).

## Checklists

### Evanioidea

#### Aulacidae

Shuckard, 1841

##### Notes

Taxonomy follows Smith (2001).

#### Aulacus

Jurine, 1807

DISPHAERON Dahlbom, 1837
AULACINUS Westwood, 1868
PAMMEGISCHIA Provancher, 1882
DISAULACINUS Kieffer, 1910
MICRAULACINUS Kieffer, 1910
NEURAULACINUS Kieffer, 1910
PARAFOENUS Kieffer, 1910
PYCNAULACUS Cushman, 1929

#### Aulacus striatus

Jurine, 1807

Aulacus arcticus Dahlbom, 1837
Aulacus exaratus Ratzeburg, 1852

##### Distribution

England.

#### Evaniidae

Latreille, 1802

##### Notes

Taxonomy follows Deans (2005).

#### Evania

Fabricius, 1775

#### Evania appendigaster

(Linnaeus, 1758)

Ichneumon appendigaster Linnaeus, 1758
Ichneumon niger (de Geer, 1773, Ichneumon)
Evania laevigata Olivier, 1792
Evania unicolor Say, 1824
Evania desjardinsi Blanchard, 1840
Evania affinis Le Guillou, 1841
Evania cubae Guérin-Méneville, 1844
Evania peringueyi Cameron, 1906

##### Notes

# Presumably an introduction to Britain as it parasitises the oothecae of non-native synanthropic cockroaches (Blattidae). Crosskey (1951) could find no locality data for British specimens and there are no relevant specimens in NHM.

#### Brachygaster

Leach, 1830

#### Brachygaster minutus

(Olivier, 1792)

Evania minuta Olivier, 1792
Evania fulvipes (Curtis, 1829, Evania)
Brachygaster rufipes Brullé, 1846 preocc.
Evania brullei (Westwood, 1851, Evania)
Brachygaster schlettereri Kieffer, 1912

##### Distribution

England.

#### Gasteruptiidae

Ashmead, 1890

##### Notes

Synonymic data mostly from Fauna Europaea.

#### Gasteruption

Latreille, 1796

FOENUS Fabricius, 1798
RHYDINOFOENUS Bradley, 1909
DOLICHOFOENUS Kieffer, 1910
TRICHOFOENUS Kieffer, 1910
PLUTOFOENUS Kieffer, 1911

#### Gasteruption assectator

(Linnaeus, 1758)

Ichneumon assectator Linnaeus, 1758
Gasteruption affectator misspelling
Ichneumon annularis (Geoffrey 1785, Ichneumon)
Foenus incertus (Cresson 1864, Foenus)
Foenus montanus (Cresson 1864, Foenus)
Foenus arca (Couper 1870, Foenus)
Foenus borealis (Thomson 1883, Foenus)
Foenus fumipennis (Thomson 1883, Foenus)
Foenus nigritarsis (Thomson 1883, Foenus)
Gasteruption nitidulum Schletterer, 1885
Gasteruption brevicauda Kieffer 1904
Gasteruption micrura Kieffer 1904
Gasteruption nevadense Kieffer 1904
Gasteruption nigropectus Kieffer, 1904
Gasteruption bakeri Kieffer 1910
Trichofoenus canadensis (Kieffer 1910, Trichofoenus)
Gasteruption abeillei Kieffer 1912
Gasteruption aberrans Strand, 1912
Trichofoenus breviterebrae (Watanabe 1934, Trichofoenus)
Gasteruption utahensis Townes 1950
Gasteruption margotae Madl 1987

##### Distribution

England, Wales, Ireland.

#### Gasteruption jaculator

(Linnaeus, 1758)

Ichneumon jaculator Linnaeus, 1758
Foenus granulithorax (Tournier, 1877, Foenus)
Foenus obliteratus (Abeille de Perrin, 1879, Foenus)
Foenus rugidorsus (Costa, 1884, Foenus)
Gasteruption thomsoni Schletterer, 1885

##### Distribution

England.

#### Gasteruption laticeps

(Tournier, 1877)

Foenus laticeps Tournier, 1877
Gasteruption foveolatum Schletterer, 1889
Gasteruption foveolum Szépligeti, 1903

#### Gasteruption minutum

(Tournier, 1877)

Foenus minutum Tournier, 1877
Foenus longigena (Thomson, 1883, Foenus)

##### Distribution

England, Wales (Alexander 1983).

#### Gasteruption pedemontanum

(Tournier, 1877)

Foenus pedemontanum Tournier, 1877
Gasteruption pedmontanum misspelling
Foenus terrestre (Tournier, 1877, Foenus)
Gasteruption trifossulatum Kieffer, 1904

##### Distribution

England.

## Supplementary Material

XML Treatment for Aulacidae

XML Treatment for Aulacus

XML Treatment for Aulacus striatus

XML Treatment for Evaniidae

XML Treatment for Evania

XML Treatment for Evania appendigaster

XML Treatment for Brachygaster

XML Treatment for Brachygaster minutus

XML Treatment for Gasteruptiidae

XML Treatment for Gasteruption

XML Treatment for Gasteruption assectator

XML Treatment for Gasteruption jaculator

XML Treatment for Gasteruption laticeps

XML Treatment for Gasteruption minutum

XML Treatment for Gasteruption pedemontanum
